# Integrated OMICs unveil the bone-marrow microenvironment in human leukemia

**DOI:** 10.1016/j.celrep.2021.109119

**Published:** 2021-05-11

**Authors:** Diana Passaro, Manuel Garcia-Albornoz, Giovanni Diana, Probir Chakravarty, Linda Ariza-McNaughton, Antoniana Batsivari, Clara Borràs-Eroles, Ander Abarrategi, Alexander Waclawiczek, Luigi Ombrato, Ilaria Malanchi, John Gribben, Dominique Bonnet

**Affiliations:** 1 Haematopoietic Stem Cell Laboratory, The Francis Crick Institute, 1 Midland Road, London NW1 1AT, UK; 2 Dynamic Neuronal Imaging Unit, Pasteur Institute, CNRS UMR, 3571 Paris, France; 3 Bioinformatic Core Unit, The Francis Crick Institute, 1 Midland Road, London NW1 1AT, UK; 4 Tumour-Host Interaction Laboratory, The Francis Crick Institute, London NW1 1AT, UK; 5 Department of Haemato-Oncology, Barts Cancer Institute, Queen Mary University of London, London EC1M 6BQ, UK

## Abstract

The bone-marrow (BM) niche is the spatial environment composed by a network of multiple stromal components regulating adult hematopoiesis. We use multi-omics and computational tools to analyze multiple BM environmental compartments and decipher their mutual interactions in the context of acute myeloid leukemia (AML) xenografts. Under homeostatic conditions, we find a considerable overlap between niche populations identified using current markers. Our analysis defines eight functional clusters of genes informing on the cellular identity and function of the different subpopulations and pointing at specific stromal interrelationships. We describe how these transcriptomic profiles change during human AML development and, by using a proximity-based molecular approach, we identify early disease onset deregulated genes in the mesenchymal compartment. Finally, we analyze the BM proteomic secretome in the presence of AML and integrate it with the transcriptome to predict signaling nodes involved in niche alteration in AML.

## Highlights

• Provide a large omics repository of the BM microenvironment of immunodeficient mice
• Demonstrate the transcriptomic overlap between different reporter niche markers
• Reveal particular pathologic crosstalk between AML and different niches
• Integrate multi-omics to predict niche biomarkers and therapeutic targets

## Graphical abstract

**Figure:**
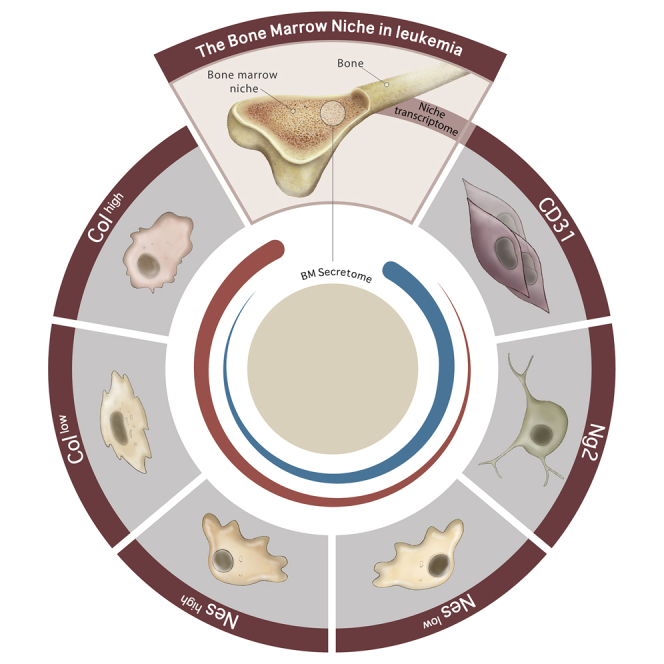

## Teaser

Passaro et al. provide a large omics repository of the BM microenvironment of immunodeficient mice. They then describe how primary human AML cells alter the molecular program of these niche components. Via integration of BM secretome and transcriptome data, they provide a repository of potential niche biomarkers.

## Introduction

Hematopoietic stem cells (HSCs) reside in a unique microenvironment referred to as the “niche,” which regulates the balance between self-renewal and differentiation of the hematopoietic stem compartment. The bone-marrow microenvironment is a complex multicellular tissue, whose non-hematopoietic component has been studied mostly in relation to its supportive function of hematopoietic lineages. Based on elegant *in vivo* depletion or expansion experiments and imaging-based approaches, a number of cell types have been suggested to contribute to the HSC niche. Studies on the bone-marrow (BM) stroma have defined individual populations in the stem cell niche regulating hematopoietic regeneration and capable of initiating leukemia (Crane and Kaeberlein, 2018; Yu and Scadden, 2016; Pinho and Frenette, 2019). To date, at least two functionally distinct perivascular niches that highly express cxcl12 and s*cf.* to dictate HSC cell fate have been identified: the arteriolar niches (Ding et al., 2012), composed mainly of arteriole-associated sympathetic nerve fibers, Nestin^high^ (Méndez-Ferrer et al., 2008) and/or Ng2^+^ cells (Yamazaki et al., 2011; Katayama et al., 2006), and the sinusoidal niches, where sinusoid-associated cxcl12-abundant reticular (CAR) cells (Omatsu et al., 2010), Nestin^low^ Lepr^+^ cells are located (Morrison and Scadden, 2014; Boulais and Frenette, 2015). These distinct vascular beds have a specific spatial distribution relative to the bone (Sivaraj and Adams, 2016) and have been shown to differentially regulate hematopoiesis and osteogenesis (Itkin et al., 2016; Langen et al., 2017; Chen et al., 2019).

Alterations of niche components has been described during the development of different blood malignancies (Kode et al., 2014; Goulard et al., 2018; Batsivari et al., 2020). Experimental models have highlighted several possibilities of crosstalk between malignant/premalignant cells and their niches as key contributors to disease initiation, progression, and resistance to therapy (Lane et al., 2009; Zhang et al., 2012; Miller et al., 2018). However, since the composition and molecular mechanisms used by the malignant niches are only partially understood, many unknowns remain.

High-throughput OMICS analyses on different interacting cellular components provide molecular insights into tissue-specific signaling networks with recent literature highlighting the high complexity of the BM microenvironment transcriptome at single-cell resolution (Tikhonova et al., 2019; Baryawno et al., 2019; Baccin et al., 2020). The BM thus represents the optimal system to apply computational tools and decipher the mutual interactions between cellular components. Here, we used genome-wide RNA sequencing, to draw a comprehensive picture of the major cell components of the BM stroma, using reporter mice widely used in BM niche research, and backcrossed in immune-deficient mice. We identified eight distinct functional clusters and unveiled their specific gene-expression signatures at steady-state hematopoiesis. We then examined the changes affecting these components during AML development using a well-established acute myeloid leukemia patient-derived xenograft (AML-PDX) model. In addition, we analyzed the BM secretome and integrated the results to the transcriptomic data to provide global mechanistic insights into changes occurring during human AML development in the niche.

## Results

### Substantial overlap among BM niche populations identified with current markers

**Figure 1:**
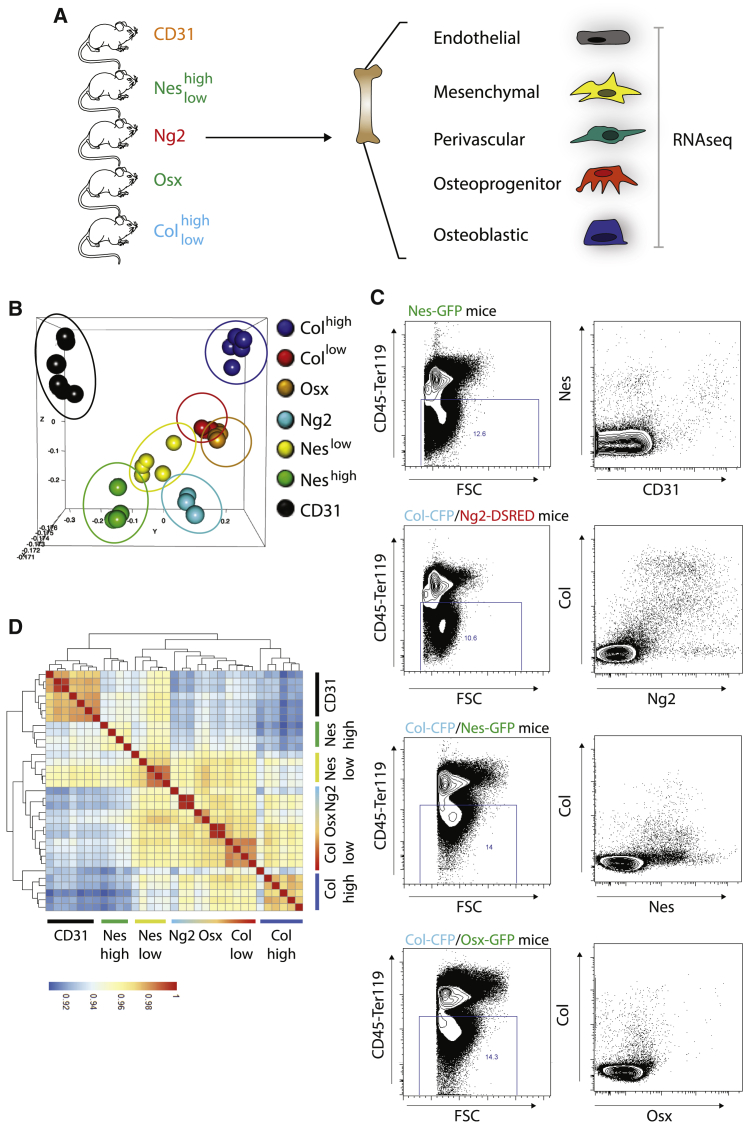
BM niche populations identified with current markers (A) Schematic figure of the experimental design depicting stromal components isolated from the BM of NSG mice carrying specific fluorescent reporters. FACS populations were analyzed by RNA-seq. CD31 n = 7; Nes^high^ n = 4; Nes^low^ n = 5; Ng2 n = 3; Osx n = 5; Col^low^ n = 4; Col^high^ n = 5. (B) 3D PCA analysis showing similarities in expression profiles between different stromal components in healthy mice. Each dot represents an experimental replicate. (C) Flow-cytometry analysis of CD45^–^Ter119^–^ BM cells derived from depicted mice showing overlap between markers associated to niche components. (D) Correlation matrix showing similarities between different stromal components in healthy mice. See also Figure S1.

To expand the current knowledge of the BM non-hematopoietic compartment, we used high-throughput RNA sequencing to perform genome-wide transcriptomic analysis of 7 sort-purified populations, by using our reporter mice for osteolineage cells (Osterix, Osx; Collagen, Col^low^ and Col^high^) and different subsets of mesenchymal cells (Ng2; Nestin, Nes^high^ and Nes^low^) and CD31 markers for endothelial cells (Figure S1A). First, we focused our attention on the molecular characterization of these stromal populations in homeostatic conditions (Figure 1A). Unbiased principal component analysis revealed tight clustering among stromal subsets (Figure 1B). Of all stromal subsets, CD31 and Col^high^ were the most transcriptionally distinct. Likewise, mesenchymal components clustered together with a certain degree of overlap between populations. Nes^high^ cells have been associated to peri-arteriolar cells (Kunisaki et al., 2013); however, their specific niche function has not been addressed. Interestingly, they show a specific transcriptional profile, only partially overlapping with Ng2 cells. A high degree of overlap is observed between Osx, classically defined as osteoprogenitors, and Col^low^ cells, while Nes^low^ population sits in the middle of the plot, suggesting a certain degree of overlap with all the aforementioned populations (Figure 1B), which we confirmed by fluorescence-activated cell sorting (FACS) analysis (Figure 1C). Correlation matrix shows the degree of similarity between populations and unveils specific cellular overlaps (Figure 1D). For instance, Nes^low^ (and to a lesser degree Nes^high^) cells partially overlap with CD31 cells, as previously reported (Figures 1C and 1D; Asada et al., 2017). Interestingly, a high degree of overlap is also observed between Ng2^+^ cells, classically considered as pericytes, and cells of the osteolineage (Figures 1C and 1D). Within the osteolineage, expression level of Col can be used to distinguish between different degrees of differentiation (Coutu et al., 2017), as suggested by the high overlap between Osx and Col^low^ (Figures 1B–1D).

### Transcriptomic signature unravels specific features of BM niche cells in homeostasis

A specific cellular taxonomy of the BM non-hematopoietic niche has been recently defined via unbiased single-cell sequencing (Baryawno et al., 2019). We could classify our 7 BM niche populations based on these defined signatures (Figure S1B), confirming their conserved function in NSG mice. However, this classification only describes the transcriptome of niche cells at relatively low depth, a limitation associated with the single-cell sequencing technology.

**Figure 2:**
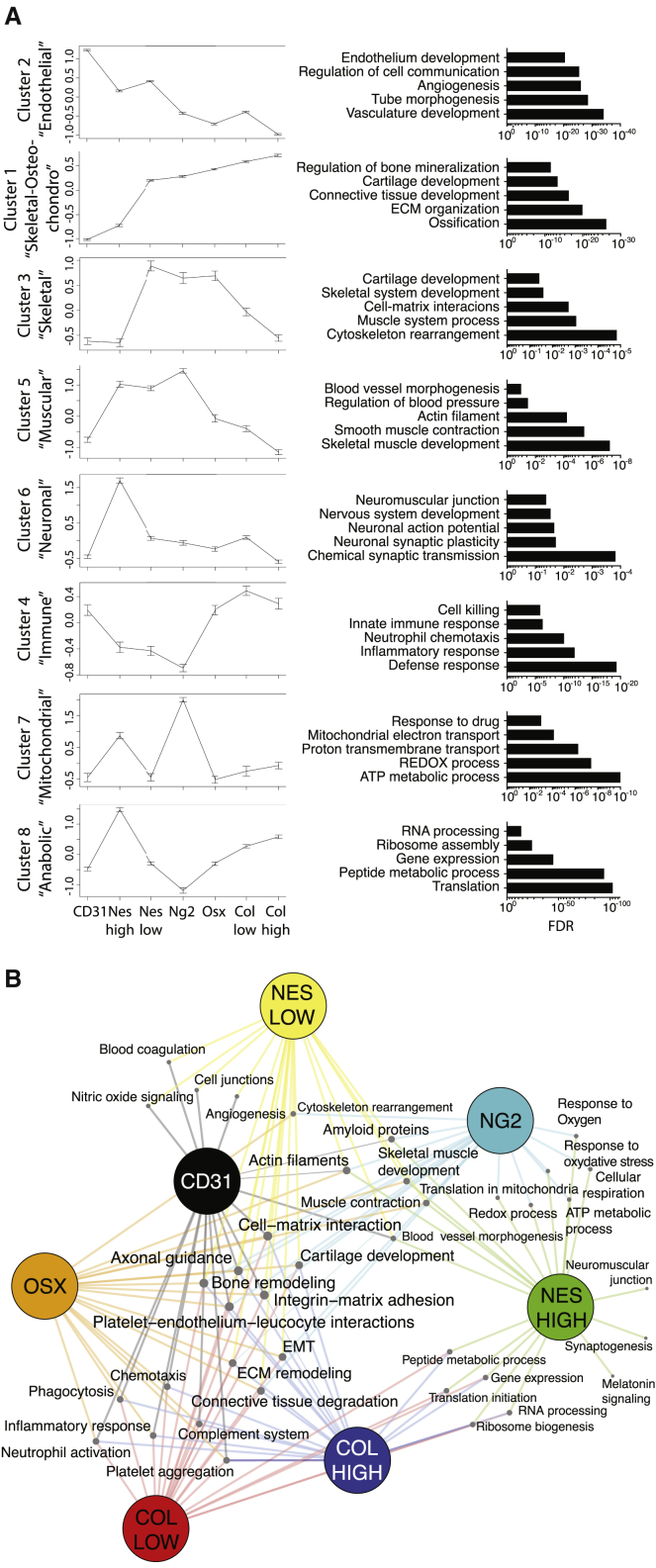
Differential clusters between BM niche populations in homeostasis (A) Cluster behaviors among niche components. Left: within-cluster averages of *Z*-scored expression profiles across stromal types from control samples (means ± SEM). Right: top enriched processes associated to each cluster. (B) Process network map showing the relatedness of the different stromal types (size of each node proportional to the number of connections). See also Figures S1 and S2 and Tables S1 and S2.

To overcome this issue and unveil thorough molecular features of BM niche cells, we used their gene-expression profiles to define 8 functional clusters of transcripts responsible of the differences between populations (Figure 2A; Table S1). Analysis of biological processes and cellular functions allowed to define specific functional features of each cluster. By combining gene-expression values of each cluster and related processes, we can recapitulate the connections between niche cells in a process network map (Figure 2B).

Cluster 2 includes genes highly expressed in CD31 cells and specific for the endothelial function, such as endothelial commitment (*pecam1*, *cdh5*, *kdr*, *emcn*, *cd34*, *flt4*, *ly6a*, *tie1*, *apln*, *pdgfb*), vessel development (*dll1*, *dll4*, *notch1*, *notch4*), regulation of angiogenesis (*eng*, *flt1*, *ephb4*, *nck1*), cell junction (*gja4*, *gja5*, *gjc1*, *cdh5*, *cldn5*, *jam2*), and platelet aggregation (*vwf*, *f2r*, *selp*, *cd36*). Further classification of the genes in this cluster can be performed by comparative analysis with recently characterized subtypes of vascular endothelial cells (type L, H and E) (Langen et al., 2017) (Figure S2A). Cluster 1 identifies a heterogeneous group of genes highly expressed in osteolineage populations (Col^high^, Clo^low^, Osx) as well as in Nes^low^ and Ng2^+^ cells, and including genes involved in the deposition and interaction with the matrix (*fap*, *CD44*, *mmp13*, *mmp16*, *tnc*, *thbs1-4*, *itgb1*), connective tissue formation (*col1a1*, *col1a2*, *dcn*, *sema3d*), chondrocytic differentiation, cartilage formation (*pth1r*, *mef2c*, *runx2*, *fbn2*), osteolineage (*alpl*, *sp7*, *runx2*, *spp1*, *smad9*, *dmp1*, *bglap1*,*2*,*3*), and bone formation (*bmp1*,*3*,*5*,*7*,*8a*). We noticed the lack of a proper mesenchymal stem cell (MSC) signature within this cluster, likely due to the low specificity of markers currently available to define such population. To dig into this question and try to isolate a cluster of genes specific to the MSC functions, we performed an analysis including gene expression of leptin-receptor^+^ (Lepr^+^) mesenchymal stromal cells from Tikhonova et al. (2019) and compared them to Nes^low^, Nes^high^, Ng2^+^ cells for their expression levels of genes in cluster 1. Interestingly, we identified two clusters of ∼300 genes similarly expressed in the Lepr^+^ and Nes^low^ cells and highly enriched in MSC signature genes (*adipoq*, *lepr*, *cxcl12*, *cxcl14*, *gpx3*, *pdgfra*, *agt*, *grem1 igfbp5*, *foxc1*, *cdh2*) (Worthley et al., 2015; Zhou et al., 2014; Zhao et al., 2019), which contains candidate markers to better define MSC population (Figure S2B; Table S2). Cluster 3 defines a small group of genes highly expressed in Nes^low^ and Ng2 involved in skeletal functions, such as muscle system processes (*acta1*, *myh1*,*4*, *myl1*, *tnnc2*, *tnnt3*), skeletal development and cell-matrix interaction (*acan*, *chad*, *col10a1*, *col271a*, *col9a1*, *lect1*), and cytoskeleton rearrangement (*tubb4a*, *acta4*, *myh11*). Cluster 5 includes genes highly expressed in Nes^high^, Ng2 and Nes^low^, involved in smooth muscle contraction (*acta2*, *myh11*, *tpm2*) and regulation of blood pressure (*acta2*, *aoc2*, *atp1a2*, *notch3*). Cluster 6 contains genes highly expressed in Nes^high^ cells, involved in neuronal-related processes, such as nervous system development (*ngfr*, *scn5a*, *egr1*, *agtr1a*, *tnfrsf12a*), synaptic plasticity and transmission (*cpeb1*, *egr1*, *syp*, *grin2a*, *pdlim4*, *prokr1*, *nos1*), and neuromuscular junction (*ank3*, *chrna1*, *musk*). Cluster 4 contains genes involved in immunoregulatory functions, such as myeloid cell differentiation and activation (*mpo*, *lcn2*, *ltf*, *cybb*, *retnlg*), neutrophil chemotaxis (*c5ar1*, *cxcl2*, *cxcr2*, *itgam*, *itgb2*, *nckap1l*, *syk*), as well as myeloid-derived suppressor cells function (*s100a8*, *s100a9*, *ngp*). This cluster is particularly high in osteolineage and endothelial cells, pointing at a specialized function for these niche components in the BM innate immunity regulation. Cluster 7 includes genes highly expressed in Nes^high^ and Ng2 cells mostly involved in mitochondrial function such as oxidative phosphorylation, ATP production and NADH metabolism (*mt-atp8*, *mt-co1*, *mt-cytb*, *mt-nd1*, *mt-nd2*, *mt-nd4*, *mt-nd5*, *mt-nd6*). Cluster 8 is particularly high in Nes^high^ and Col^high^ cells and mainly contains genes involved in cellular anabolic processes, such as RNA processing and translation, ribosome assembly, and peptide metabolism (38 *rpl* and 31 *rps* genes, *eif3h*, *eef1a1*, *uba*, *ubb*, *snrpa*, *naca*) (Figures 2A and 2B). Overall, our clusters analysis allowed the identification of groups of genes/functions defining unique features as well as shared properties among niche populations.

### Human AML alters the homeostatic functional identity of niche cells

**Figure 3:**
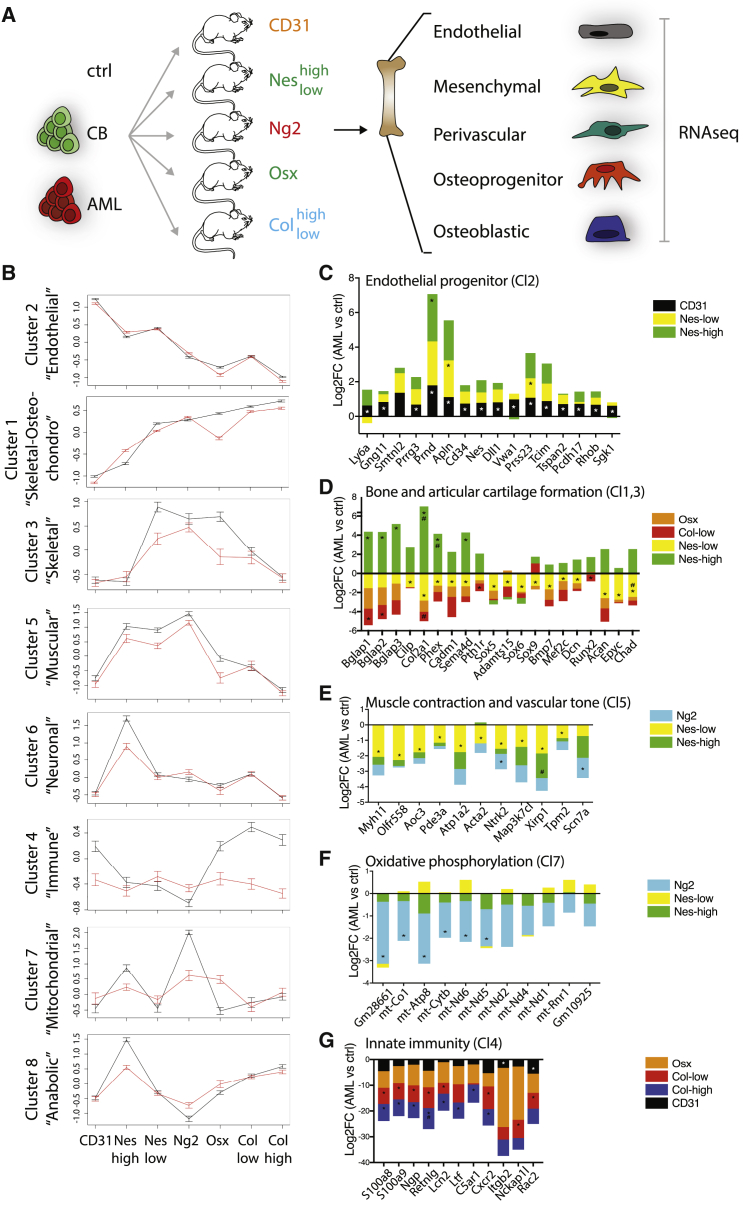
Human AML impact on the functional identity of the BM niche (A) Schematic representation of the experimental setup. Depicted stromal components were isolated from the BM of NSG mice carrying specific fluorescent reporters and non-transplanted (ctrl) or xenografted with healthy hematopoietic cells (CB) or patient-derived AML samples (AML; see Table 1 for details). FACS-sorted populations were analyzed by RNA-seq. CD31: ctrl n = 7, CB = 4, AML = 15; Col^high^: ctrl n = 5, CB = 2, AML = 9; Col^low^: ctrl n = 4, CB = 3, AML = 13; Nes^high^: ctrl n = 4, CB = 2, AML = 11; Nes^low^: ctrl n = 5, CB = 2, AML = 11; Ng2: ctrl n = 3, CB = 3, AML = 12; Osx: ctrl n = 5, CB = 4, AML = 10. (B) Comparison of within-cluster averages of *Z*-scored expression profiles between control samples (black) and leukemic samples (red). (C–G) Deregulation (mean log2FC) of genes in depicted clusters in the presence of AML (^∗^means FDR <0.1). See also Figures S3 and S4 and Table S3.

We next analyzed how the presence of human AML engraftment impacts on the molecular signature of BM niche components. We used several donor BM patients representing different subgroups of the human AML disease spectrum (Table 1) as well as human AML cell lines and healthy human hematopoietic cells (CB) to engraft unconditioned immunodeficient mice, and isolated BM stromal cells as depicted in Figure 3A. t-distributed stochastic neighbor embedding (tSNE) analysis showed that AML does not impact on the global cellular identity of these niche components, since most of the replicates of each population reside in confined groups irrespectively of belonging to a healthy or BM with AML (Figure S3A). We used our previously described clusters to unravel the AML impact on BM niche function. Some of the clusters remain stable upon AML engraftment, while others lose their expression trend (Figures 3B and S3B).

**Table 1:** AML patient-derived samples

Sample ID	Karyotype	FAB	Age	Sex	NPM	FLT3
AML1^a^	Inv(3)(Q21Q26)/t(8;13)(p21)	M0	39	M	WT	WT
AML2^b^	trisomy 13	M1	67	F	Mut	n.a.
AML3^a^^,^^b^	normal	M2	72	F	Mut	WT
AML4^a^	T(6;11)(Q27;Q23)	M5a	36	M	WT	WT
AML5^a^^,^^b^	normal	M4	24	F	WT	ITD
AML6^b^	Del7q+8+11q	M4	61	F	WT	WT
AML7^a^	1q	M5a	53	M	Mut	WT
AML8^b^	normal	M1	61	M	Mut	ITD

Patient-derived AML samples used in the study and their genetic and clinical characteristics. n.a., not applicable.

a Samples used for transcriptome experiments.

b Samples used for secretome experiments.

The overall signature of cluster 2 remains stable. However, we can identify changes in expression of specific subsets of genes, particularly the one associated with endothelial progenitor signature (Figure 3C). In fact, most of the upregulated transcripts correspond to genes highly expressed in type E cells, defined as progenitor/immature endothelial cells involved into matrix remodeling as well as angiogenesis and osteogenesis upon radiation injuries (Langen et al., 2017; Chen et al., 2019; Figure S3C). We also notice signs of endothelial-to-mesenchymal transition (EnMT; Figure S3D), a process already observed in myelofibrosis (Erba et al., 2017) and downregulation of genes involved in hemostasis (Figure S3E), suggesting a potential role of the endothelial component in severe complications of AML disease such as hemorrhages (Balmages et al., 2018).

Skeletal processes involved in bone and cartilage formation appear affected by AML, as shown by downregulation of specific genes in cluster 1 and 3 in Nes^low^ and Col^low^ cells (Figure 3D). Interestingly, the same genes are instead upregulated in Nes^high^ cells, pointing at a potential compensation mechanism (Figure 3D). In line with the EnMT phenotype, matrix and bone remodeling genes are also upregulated in CD31 cells (Figure S3F). The neurogenesis function seems downregulated in most of the mesenchymal cells (Nes^low^, Nes^high^, Ng2), while some specific genes are upregulated in CD31 cells (Figure S3G).

Another process highly affected by the presence of AML is the one associated with the smooth muscular function, as shown by downregulation of genes of cluster 5 in Nes^low^, Nes^high^, and Ng2 involved in muscle contraction and vascular tone regulation (Figure 3E).

On the same line, most of the genes of cluster 7 are downregulated in Ng2 and Nes^high^ cells (Figure 3F), pointing at a decreased metabolic function in muscular cells.

Finally, the BM innate immunity regulation function in cluster 4 is massively downmodulated in the presence of AML, both in the osteolineage and endothelial cells (Figure 3G).

Parallel to cluster-tailored analysis, we also performed an unbiased analysis of the global transcriptome deregulation of leukemic BM niche components. We used the top altered genes in each stromal type (Table S3) to reveal specific deregulation patterns estimated as hidden features from the data (Figure S4A). These deregulation patterns were either specific or shared across cell types, uncovering potential stromal relationships in the context of AML. We build a global map highlighting the top deregulated processes and their behavior across stromal types (Figure S4B). Notably, we find shared patterns across osteolineage and endothelial cells in terms of downregulated processes, such as ion transport, cell adhesion, and immune regulation. Interestingly, while the process of vascular morphogenesis is similarly downregulated in both endothelial and osteolineage niche components, the pro-angiogenic response is specifically upregulated in the endothelial compartment and downregulated in the osteolineage, suggesting different processes driven by the two stroma cell types. Similarly, we evidence several common clusters of upregulated processes across the mesenchymal components, including a pro-proliferative response, cell-matrix interaction, connective tissue degradation, and specific signaling pathways such as Notch, NfkB, Esr1, and Wnt. Overall, our analysis shows multiple injuries to the homeostatic balance upon human AML engraftment in the BM niche specific for a single cell type or involving multiple components and potentially associated to AML pathologic phenotypes (Figure S4B).

### Transcriptomic analysis at early time points unveils the progressive detriment of the BM mesenchymal niche in AML

**Figure 4:**
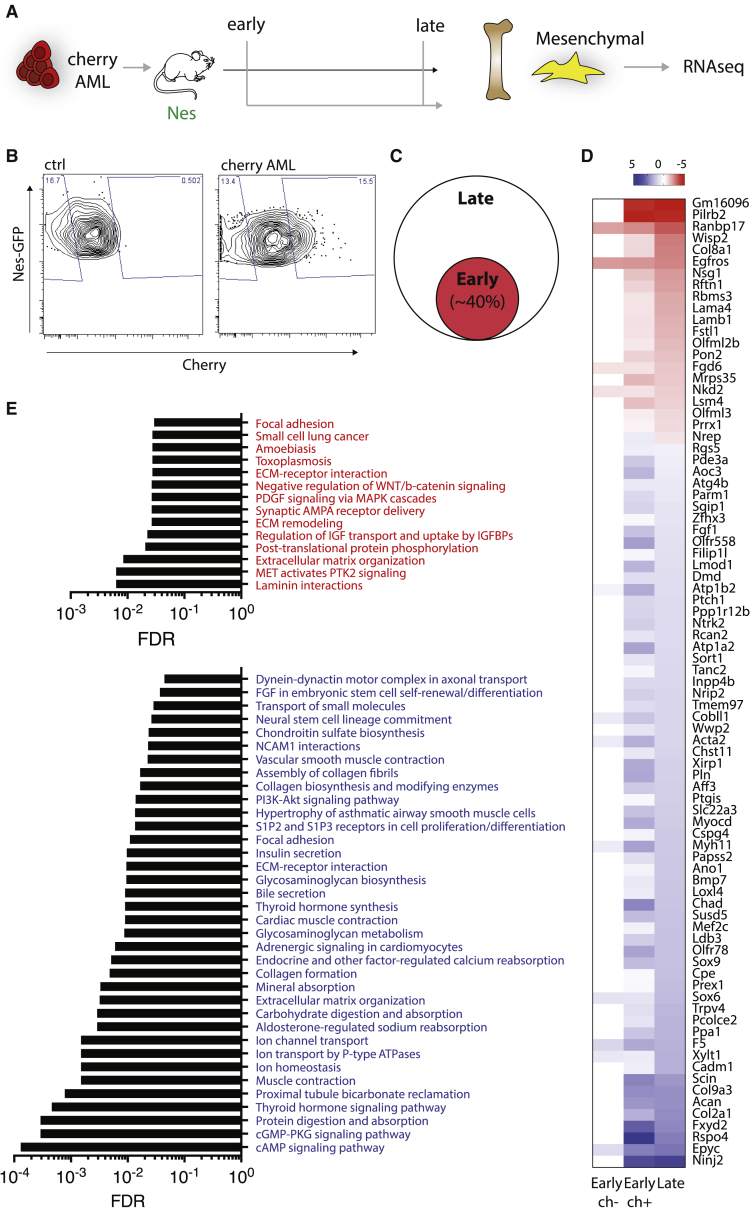
Progressive detriment of the BM mesenchymal niche in AML (A) Schematic representation of the experimental setup. Nes^low^ mesenchymal stromal cells were isolated from the BM of Nestin^GFP^-NSG mice xenografted with human cherry^+^-AML cell lines at early and late time points of leukemic engraftment. FACS-sorted populations were analyzed by RNA-seq. Ctrl n = 5; AML early ch^+^ n = 6; AML early ch^–^ n = 6; AML late n = 6. (B) Flow cytometry profile of Nes^low^ cells from control mice or mice engrafted with human cherry^+^-AML cell lines at an early time point. (C) Pie chart showing the proportion of late deregulated genes also deregulated at early time points. (D) Heatmap showing the top transcripts deregulated in both the early and late stage of leukemic development in the mesenchymal niche. (E) Top upregulated (red) and downregulated (blue) processes in the early stage of leukemic engraftment in the mesenchymal niche. See also Figure S5.

Our data shed light on global molecular changes of the BM niche in the presence of fully established leukemia, in a disease setting mimicking the clinical scenario of patients at diagnosis. However, little is known about the dynamics of niche deregulation in the BM at early disease onset. To investigate whether any of the observed molecular changes could be detectable during the early onset of the disease, we used a recently developed system allowing identification and isolation of niche cells in close proximity with leukemia via transfer of a soluble fluorescent lipid-permeable mCherry (sLP-mCerry) marker (Ombrato et al., 2019, 2021), thus allowing molecular studies of the niche at early stage of disease. We focused our attention on the most abundant component of the niche, Nestin^+^ mesenchymal cells (Nes^low^), due to the challenging low number of retrieved niche cells at early time points. Upon engraftment with human leukemic cells expressing the sLP-mCerry, we isolated cherry^+^ Nes^low^ (ch^+^) labeled early leukemic niche cells, and cherry^–^ Nes^low^ (ch^–^) cells present in the portion of the same BM, but not in contact with leukemic cells (Figures 4A and 4B). We compared the transcriptomic profile of ch^+^ Nes^low^ cells from the early niche (1%–10% engraftment, “early”) and Nes^low^ cells from heavily engrafted mice (70%–80% engraftment, “late”), a time point where all cells are in contact with leukemic cells. We observed that a big proportion of the AML-dependent molecular changes highlighted at a late time point were already present in the early niche. For instance, 40% of the genes deregulated in late Nes^low^ cells were also significantly deregulated in early ch^+^ Nes^low^ cells (Figures 4C, 4D, and S5), while only the 0.05% of them were altered in ch^–^ Nes^low^ cells (Figure 4D). This highlights the great potential of this approach to achieve specific physical isolation of the early niche in contact with AML cells. Our analysis allowed the identification of a large set of genes and pathways (Figures 4D, 4E, and S5) deregulated in the early crosstalk between mesenchymal cells and leukemia, which represent strong candidates as potential biomarkers for early AML diagnosis or targets for the rescue of the pathological phenotypes associated with the mesenchymal niche.

### The BM secretome of patient-xenografts provides a reservoir of potential direct mediators of AML pathogenesis

**Figure 5:**
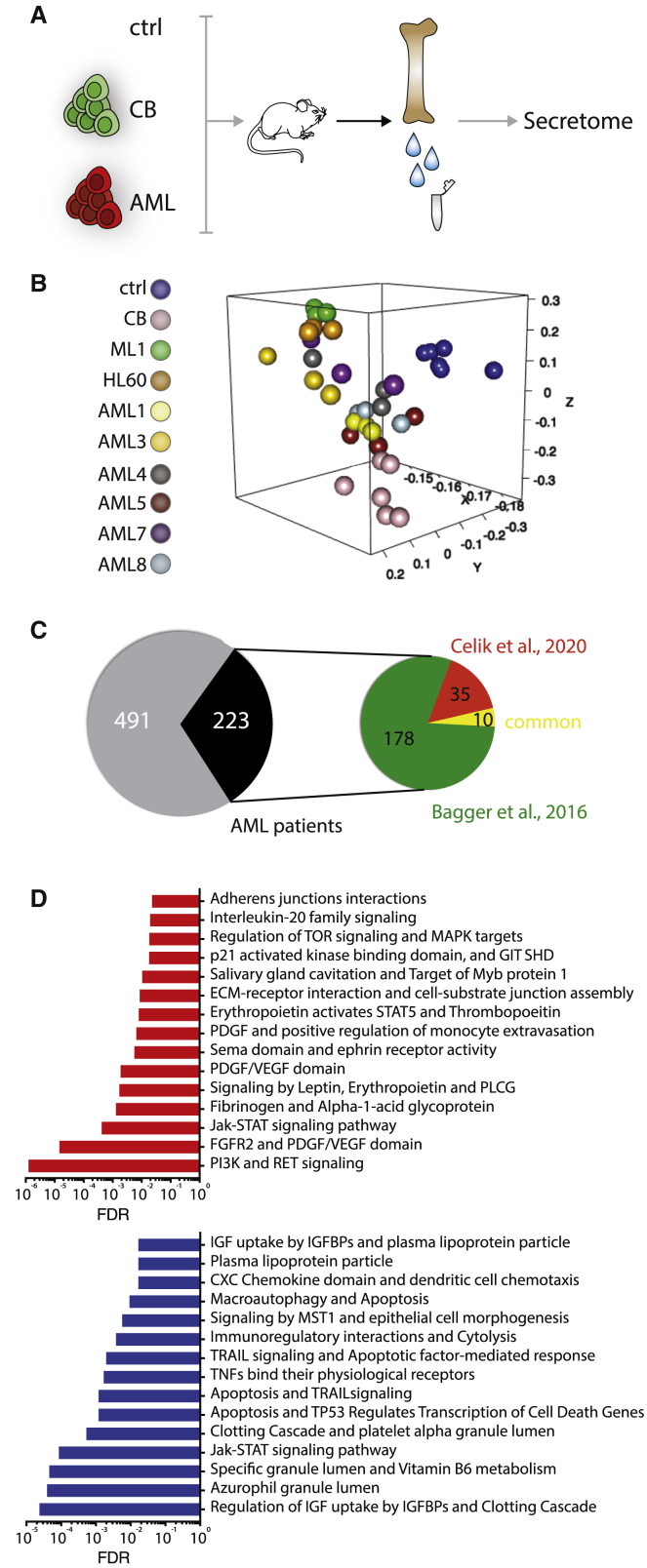
The BM secretome in AML patient xenografts (A) Schematic representation of the experimental design. BM extracellular proteins were isolated from NSG mice non-transplanted (ctrl) or xenografted with healthy hematopoietic cells (CB) or human AML (AML; see Table 1 for details), and BM secretome was analyzed on a SOMAscan array. (B) 3D PCA showing similarities in protein expression profiles between samples. Each dot represents an experimental replicate. (C) Pie chart showing the number of deregulated proteins validated with comparison with depicted human datasets. (D) Top processes specifically upregulated (red) and downregulated (blue) in AML xenografts BM secretome compared to ctrl and validated on human data. See also Tables S4 and S5.

Our transcriptomic data unravel molecular insights into the response of the BM niche to the presence of AML, but how much of this response derives from a direct crosstalk is unclear. To gain insight into changes in the BM milieu upon human AML engraftment, we performed a proteomic analysis of the BM secretome in AML xenografts (Figure 5A). Although we observed a certain degree of heterogeneity among AML samples, possibly linked to genetic diversity in our cohort (Figure 5B; Table 1), we identified a large common secretome signature within the AML cohort (Tables S4 and S5). We focused our attention on the top commonly deregulated proteins and processes and validated their translational impact by comparing our dataset to recently published proteomics data retrieved from healthy donors and AML patients, as well as to a large cohort of patient-derived transcriptomic dataset (Bagger et al., 2016; Çelik et al., 2020). We observed a broad correspondence between xenograft and human patients’ data, with more than 30% overlap (Figure 5C; Table S4). Among the top deregulated pathways validated with human data, we found as expected AML associated signaling such as the RAF/MEK/ERK and PI3K/AKT cascades; interestingly, we observed upregulation of proteins involved in the RET signaling (Epiregulin, Gfra-1, Shp-2), recently highlighted as a mediator of normal HSC maintenance and expansion (Grey et al., 2020). We also found increased production of proteins involved in the interaction with the extracellular matrix (Osteonectin, Thrombospondin-1, Fibronectin, a1-Antitrypsin). The downmodulated pathways are largely related to the immune function, with decreased dendritic cell chemotaxis (Cxcl5, Cxcl9, Ccl5), immunoregulatory function and cytolysis (Granzyme H, MICB, Granulysin, Ilt-2, CD48). Interestingly, the clotting cascade and platelet activation pathways are also downregulated (Tfpi, Dtk, KLKB1, Prekallikrein, a2-Antiplasmin, Coagulation Factor X; Figure 5D; Table S4).

### Integration of niche transcriptome and BM secretome predicts molecular nodes involved in niche alteration in AML

**Figure 6:**
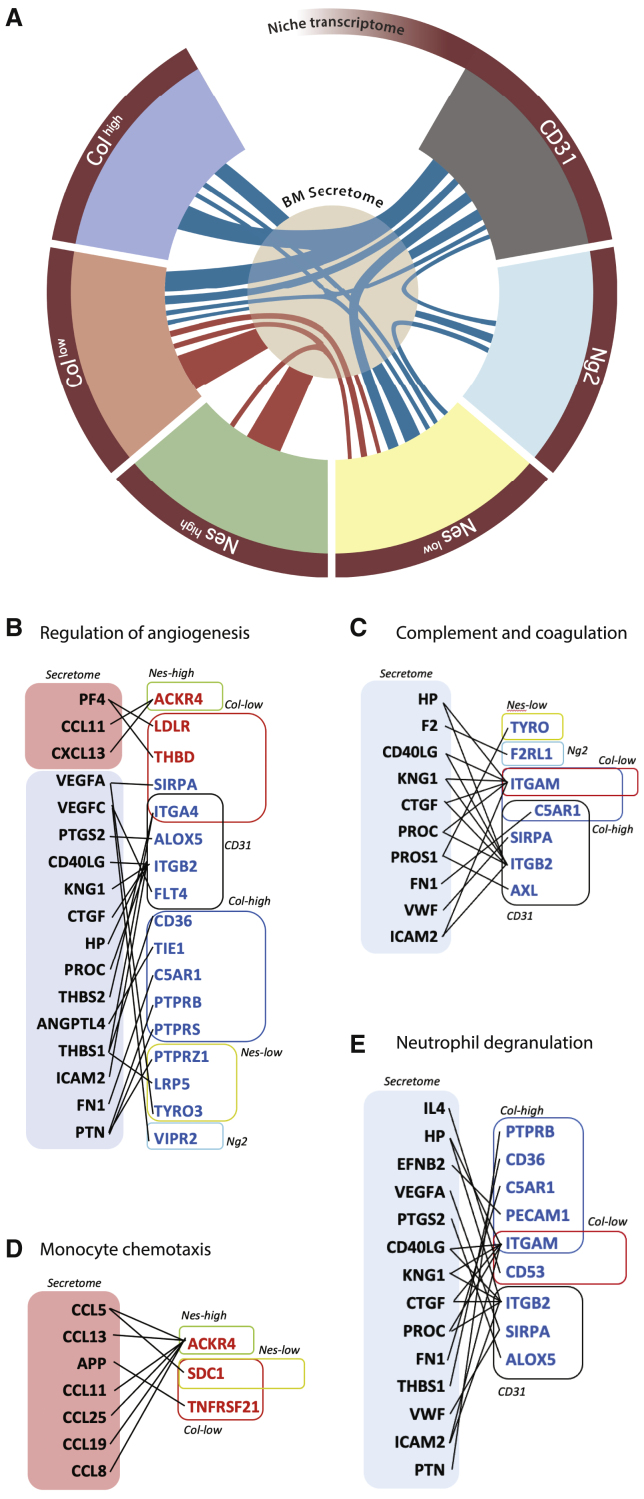
Integration of niche transcriptome and BM secretome (A) Graphic map of the ligand-receptor interactome between distinct niche components and the BM secretome. The thickness of the connecting lines is proportional to the number of interactions. (B–E) Sketch graphs of upregulated (red) and downregulated (blue) predicted ligand-receptor interactions between BM secretome and niche transcriptome. See also Figure S6 and dynamic figure https://giovannidiana.github.io/integratedomics/

To predict direct interactions responsible of the BM niche and AML crosstalk, we integrated the secretome and transcriptome data. Ligand-receptor analysis allowed the identification of upregulated and downregulated nodes of interaction between the BM *milieu* and niche components (Figure S6). The interactome showing ligand-receptor pairs involved in specific pathways is summarized in Figure 6A and can be explored at https://giovannidiana.github.io/integratedomics/. Overall, we noticed a peculiar dual distribution of signaling pathways between the osteo-endothelial niche and the mesenchymal components (Figure 6A). Among the others, we noticed an interesting combination of pro- and anti-angiogenic cues associated to multiple stromal types, suggesting distinct local regulation (Figure 6B). Interestingly, several signaling mediators of the complement and coagulation cascade as well as platelet activation and hemostasis are downregulated (Figure 6C), in line with clinical reports pointing at bleeding disorders and thrombocytopenia in AML patients (Del Principe et al., 2017; Balmages et al., 2018; Ahrari et al., 2019; Just Vinholt et al., 2019). On the immune side, increase in chemokines involved in monocyte chemotaxis is predicted to interact with the deregulated mesenchymal niche (Figure 6D), while reduced mediators associated to neutrophil function are connected to the osteo-endothelial niche (Figure 6E), the compartment mostly involved in the regulation of innate immunity under homeostasis according to our cluster analysis (Figure 2A, 3B, and 3G). In conclusion, our integrated sequencing data with the BM secretome of AML xenografts allowed the identification of specific signaling nodes as candidate mediators of stromal alteration in human AML.

## Discussion

The BM microenvironment has gathered increasing attention over the last years. Far from being a simple monolayer of supportive cells, the “hematopoietic niche” has revealed a high degree of complexity when studied *in vivo* with functional genetics (Crane et al., 2017; Wei and Frenette, 2018). Several markers have been proposed to label specific supportive niches within the tissue. Nevertheless, the high degree of overlap between these markers have only recently been evidenced by us (Figures 1 and S1) and other groups in the field (Coutu et al., 2017). The advent of high-throughput sequencing techniques now allows the molecular characterization of these cells with unprecedented precision (Tikhonova et al., 2019; Baryawno et al., 2019; Baccin et al., 2020). Our data covering the transcriptome of the bone-marrow niche at high sequencing depth contribute to fill these gaps by providing a large repository of potential markers to better define specific cell populations (Figures 2 and S2). Among the others, of high interest is the Nes^high^ population, known for a long time but not studied in detail. Despite sharing some features with the other cell of mesenchymal origin, these cells appear strikingly different at the molecular level under homeostatic condition, highly expressing genes involved in RNA processing and translation (Figures 1 and 2). This peculiar feature could explain the ability of Nes^high^ cells to rapidly adapt to a modified environment in the presence of leukemia and overcome the disrupted functions of other cell components (Figure 3), thus opening new perspective to deepen our knowledge on the nature and function of these cells. Moreover, we have also evidenced a remarkable association between the osteolineage and endothelial compartments, sharing specific functions in the regulation of tissue homeostasis and responding to leukemia insult as a highly connected unit (Figures 2, 3, and 6). A tight proximity between a specific type of endothelial cells and the bone surface has been previously evidenced, with a prominent role in regulating angiogenesis, osteogenesis, and matrix remodeling (Kusumbe et al., 2014; Langen et al., 2017). Our analysis allowed the prediction of the local mutual interaction between these two compartments, further identifying a potential role in the regulation of innate immunity.

The presence of AML dramatically impacts on niche homeostatic function, as shown by our cluster-tailored analysis. Moreover, we highlighted that a pathologic crosstalk exists not only between AML and different niche cells but also within niche fractions. These intra-stroma interactions add another layer of complexity but also new potentials for drug interventions. The rebound of this extensive tissue deregulation on leukemia progression and normal hematopoiesis has only started to be addressed (Passaro et al., 2017; Duarte et al., 2018), and our data open novel paths for further studies of great relevance to the field. At our knowledge, no methods are available to date to isolate the living leukemic niche at single-cell resolution and at early time points of the disease. Laser-microdissection (Pacheco et al., 2018) or pipette aspiration (Silberstein et al., 2016) methods in bones have proved to have several limitations, notably poor resolution, age constraints, as well as the requirement for complex systems and technologies. The painting tool we have used (Figures 4 and S5) was designed to detect the early metastatic niche in lung cancer with wide-range flow cytometry techniques (Ombrato et al., 2019), and we have proved its applicability to isolate and study living hematopoietic niche cells at an early stage of leukemia dissemination in comparison to full-blown disease, producing data with great potential of unravel early mechanisms responsible for the progressive alteration of the leukemic niche.

The data presented in this work provided a large omics repository of the environment of immunodeficient mice. Despite the profound immunological defects of this model, it is widely used to produce impactful insights in the field of tumor xenografts, including the leukemic microenvironment (Abarrategi et al., 2018; Medyouf, 2017). Moreover, our results showing no major differences between our niche components and the functional clusters defined in immunocompetent mice in homeostasis (Tikhonova et al., 2019; Baryawno et al., 2019; Figure S1) as well as similarity between the deregulated proteomic signature in our xenotransplant model to the one reported in AML patients (Figure 5) endorse the validity of the xenograft model to study the non-hematopoietic BM microenvironment. Nevertheless, we recognize a certain number of limitations, notably the lack of the adaptive immune system. While the impact of AML on the immune environment has been recently addressed in patients (van Galen et al., 2019), very little is known about the non-hematopoietic niche at the molecular level in the human BM. It remains thus necessary to move these molecular studies a step forward in the translation process, by sequencing the human BM niche in its entirety and identifying specific markers to label functional environmental components in the regulation of normal and malignant hematopoiesis.

AML is a dreadful disease and in most cases is extremely difficult to treat in an efficient manner. There is a constant effort from the scientific community to try and identify novel therapeutic strategies tailored on specific subgroups, which offer promising results (Konopleva et al., 2016; Tomlinson et al., 2019; Perl, 2019; Roboz et al., 2020). We and others (Passaro et al., 2017; Duarte et al., 2018; Guarnerio et al., 2018; Battula et al., 2017; Tabe et al., 2017) have provided strong evidence that the microenvironment is an additional active part of the leukemic unit. Thus, our approach analyzing the bone-marrow environment in response to AML patients’ samples derived from different subgroups allows the identification of common pathologic hallmarks of the niche in AML disease, with a great potential to serve as a repository of biomarkers and therapeutic avenues. Further studies using similar approaches could follow to dive into differences between AML subgroups and draw subgroup-tailored deregulation maps in the microenvironment, which could serve as potential targets for personalized medicine.

## STAR★Methods

### Key resources table

**Table:** 

REAGENT OR RESOURCE	SOURCE	IDENTIFIER
**Antibodies**		

CD45 30-F11 1 in 400 Mouse	eBioscience	45-0451-82
TER119 Ter-119 1 in 400 Mouse	eBioscience	12-5921-82
CD31 390 1 in 400 Mouse	eBioscience	25-0311-82
EasySep Human CD34 Positive Selection kit	StemCell Technologies	17856
EasySep Human CD45 Positive Selection kit	StemCell Technologies	100-0105
EasySep Mouse CD45 Positive Selection kit	StemCell Technologies	18945
InVivoMAb anti-human CD3	Euromedex/ Bioxcell	BE0001-2

**Bacterial and virus strains**		

sLP–mCherry pRRL lentiviral backbone	Ombrato et al., 2019, 2021	N/A

**Biological Samples**		

See Table 1	N/A	N/A

**Chemicals, peptides, and recombinant proteins**		

DNase I	Sigma-Aldrich	D4527
Collagenase	Sigma-Aldrich	C0130
Dispase II	Sigma-Aldrich	D4693
Heparin	Sigma-Aldrich	H3149

**Critical commercial assays**		

SomaScan® Platform	Somalogic	https://lifesciences.somalogic.com/technology
Ovation® RNA-Seq System V2 kit (part No. 7102	Nugen	M01206 v9
RNeasy Mini kit	QIAGEN	74106

**Deposited data**		

RNA-sequencing data	Geo Bank	GSE148626
Secretome data	N/A	Table S5

**Experimental models: Cell lines**		

HL60	ATCC	Cell Service, The Francis Crick Institute
ML1	ATCC	Cell Service, The Francis Crick Institute

**Experimental models: Organisms/strains**		

NOD-SCID IL2Rg^null^ (NSG)	Jackson Laboratory	005557
NSG-NESTIN-EGFP	The original strain is a kind gift from Dr G. Enikolopov. Backcrossed to NSG background at the BRF, The Francis Crick Institute (generation 8)	N/A
NSG-NG2-DSRED	Original strain from Jackson Laboratory (008241). Backcrossed to NSG background at the BRF, The Francis Crick Institute (generation 8)	N/A
NSG-COL2.3-CFP	The original strain is a kind gift from Dr DW Rowe. Backcrossed to NSG background at the BRF, The Francis Crick Institute (generation 8)	N/A
NSG-OSX-CRE-GFP	Original strain from Jackson Laboratory (006361). Backcrossed to NSG background at the BRF, The Francis Crick Institute (generation 8)	N/A

**Software and algorithms**		

FACSDiva	BD	N/A
FlowJo	FlowJo	Version 9.9
CASAVA BCL to FastQ	N/A	Version 2.16
RSEM package	N/A	Version 1.2.29
STAR alignment algorithm	N/A	Version 2.5.1b
DESeq2 package	N/A	Version 1.22.2
R programming environment	N/A	Version 3.2.3
Bioplotr package	N/A	N/A
prcomp function	N/A	N/A
plot3d function from the rgl package	N/A	N/A
Pheatmap package on R	N/A	N/A
“limma” package	N/A	N/A
Density-based algorithm	Rodriguez and Laio, 2014	N/A
Model-based Bayesian method	Adapted from Diana et al., 2019	N/A
Dynamic figure of ligand-receptor interaction	https://github.com/giovannidiana/integratedomics	https://giovannidiana.github.io/integratedomics/

### Resource availability

#### Lead contact

Please contact Prof Dominique Bonnet, The Francis Crick Institute, Haematopoietic Stem cell laboratory, 1 Midland Road, NW1 1AT, London, UK; dominique.bonnet@crick.ac.uk.

#### Materials availability

This study did not generate new unique reagents.

#### Data and code availability

The accession number for the transcriptome sequencing data generated in this study are deposited on Geo bank (reference number: GSE148626). Protein array raw data are available in Table S5.

### Experimental model and subject details

#### Human primary samples

Umbilical Cord Blood (CB) samples were obtained from normal full-term deliveries after signed informed consent. AML samples used for xenografts were obtained after informed consent at St Bartholomew’s Hospital (London, UK). Details are listed in Table 1. The collection and use of all human samples were approved by the East London Research Ethical Committee (REC:06/Q0604/110) and in accordance with the Declaration of Helsinki. Mononuclear cells (MNCs) were isolated by centrifugation using Ficoll-Paque TM PLUS (GE Healthcare Life Sciences, Buckinghamshire, UK). For CB samples, the cells were processed within 24 hours following collection. Cells were enriched for CD34+, using an EasySep Human CD34 Positive Selection kit (StemCell Technologies, Vancouver, Canada) according to the manufacturer’s instructions, with a purity of 85 to 99%. T cells were depleted from all AML samples using the anti-CD3 mAb OKT-3 (West Lebanon, USA).

#### Mouse models

All animal experiments were performed under the project license (PPL 70/8904) approved by the Home Office of UK and in accordance to The Francis Crick institute animal ethics committee guidelines. Nestin-GFP (Mignone et al., 2004) mice were a kind gift from Dr G. Enikolopov. Col2.3-CFP (Visnjic et al., 2001) mice were a kind gift from Dr. DW Rowe. Osx-cre-Gfp (Rodda and McMahon, 2006) and Ng2-Dsred (Zhu et al., 2008) strains were purchased from Jackson laboratory. NOD-SCID IL2Rgnull (NSG) strains were also obtained from Jackson Laboratory, Bar Harbor, Maine, USA) and were bred at The Francis Crick Institute Biological Resources Facility in individually vented cages (IVCs) under Specific Pathogen Free (SPF) conditions. All reporter strains were backcrossed into the NSG background (generation 8 or more) at The Francis Crick Institute.

#### Cell lines

HL60 and ML1 cells were grown in RPMI1640. All cell lines were tested for mycoplasma prior to commencing experiments. HL60 came originally from ATCC (distributor LGC standards, UK), ML1 from European Collection of Authenticated Cell Cultures (ECACC) and were grown by our cell service at the Institute. Before using these lines, they were authenticated using the Short Tandem Repeat (SRF) profiling. All media were supplemented with 10% FBS and 1x Penicillin-Streptomycin and all reagents were from GIBCO®-Life Technologies (Paisley, UK). The sLP–mCherry sequence was produced and cloned into a pRRL lentiviral backbone as previously described (Ombrato et al., Nature 2019). Briefly, a soluble peptide (SP) and a modified TAT peptide were cloned upstream of the mCherry cDNA, under the control of a mouse PGK promoter. HL60 and ML1 cells were stably infected with sLP–mCherry lentiviral particles (MOI 10) and flow sorted 48 hours later to select the positive cells.

### Method details

#### AML and HSPCs transplantation assay *in vivo*

For xenografts assays, human AML cell lines (HL60, ML1; 2x10^6^), Okt3-treated AML patient-derived samples (2-8x10^6^) and healthy HSPC (1x10^6^) were transplanted into 8 to 12 week old unconditioned mice by i.v. injection. Each experimental cohort contained an equal number of male and female mice. BM engraftment was assessed by BM aspirate. Mice were euthanized when human engraftment was > 50% (or 1%–10% for early time point experiments), with an age of 13 to 20 weeks.

#### Bone marrow cell isolation

At the end of each experiment, animals were euthanized by cervical dislocation and the 6 rear bones collected in cold PBS. To retrieve BM niche cells, bones were dissected in small pieces of 2-3 mm diameter and incubated 20 min at 37 degrees in a HBSS digestion solution containing 2% FBS, DNase I (10 μg/ml), Collagenase (1 mg/ml), Dispase II (5 mg/ml) and Heparin (20 U/ml), all from Sigma-Aldrich, Gillingham, Dorset, UK. Bone pieces were next crushed with mortar and pestle in the same digestion solution and incubated at 37 degrees for 20 min. Cell suspension was then homogenized with a micropipette and filtered with a 100μ cell strainer. To retrieve BM extracellular proteins, the BM homogenized suspension was flushed into 100 ul PBS and spin at 300 g. Supernatant was collected and kept at −80 degrees until analysis.

#### Flow cytometry analysis and cell sorting

Flow cytometry analysis was performed using a Fortessa flow cytometer (BD Biosciences, Oxford, UK). Dead cells and debris were excluded from the analysis using 4,6, diamidino-2-phenylindole (DAPI) or SYTOX Green (ThermoFisher) staining. Cell sorting was performed using a FACS Aria SORP (BD Biosciences, Oxford, UK). Data were analyzed by FACSDiva (BD) or FlowJo (version 9.9) software. To sort BM niche cells, human and murine hematopoietic cells were excluded using an EasySep Human CD45 Positive Selection kit and a murine CD45 positive selection kit (Stem Cell Technologies, Vancouver, Canada) according to the manufacturer’s instructions, with a purity of 85 to 99%. Remaining cells were stained with hCD45 (2B11), mCD45 (30-F11), Ter119 (Ter-119) and mCD31 (390), all from eBioscience, and specific stromal cells were sorted in RNAeasy RTL buffer (RNeasy Mini kit, QIAGEN) based on the expression of CD31 or specific fluorescent reporters (GFP for Nestin and Osx; DSRED for Ng2; CFP for Col2.3; Cherry for labeled niche). Flow cytometry plots displayed in this manuscript represent one set of data points from at least 3 replicates.

#### Sample preparation for RNA-sequencing

RNA was extracted using RNeasy Mini kit (QIAGEN) following manufacturer instructions. The quality and concentration of total RNA were determined on Agilent 2100 Bioanalyzer using the Eukaryote Total RNA Pico Assay. Most of the total RNA has average RIN number 5-8, with a concentration at least 4 pg/μl. Some of the samples were concentrated on a speedvac without heat to obtain a final volume of 5 μl. 3.5 ng of Total RNA in 5 μL volume was used to generate cDNA synthesis with Nugen Ovation® RNA-Seq System V2 kit (part No. 7102). The resulting SPIA-cDNA were normalized to 100 ng in 15 μL based on Qubit DNA HS assay. Fragmentation was done using 8 microTUBE-15 AFA Beads Strip V2 (PN 520159) on Covaris E-series at setting 20%DF, 18W, 200 burst, 20C tempt, 120 s treatment time and no Intensifier. Only 10-30 ng in 10 μL of fragmented cDNA was used starting from the End Repair step of the Ovation® Ultralow Library Systems V2 (part No. 0344NB) protocol, with 10 cycles PCR. The RNaseq libraries were quality checked on Qubit DNA HS assay, Bioanalyser and Illumina Ecoreal QPCR followed by Illumina PE51 sequencing on Hiseq 2500 V3 reagents.

#### Samples preparation for secretome

Supernatants from processed bones were spin down to remove precipitates and cellular debris. Protein concentration was quantified using Bradford assay and quality assessed via gel electrophoresis. 20 μg of proteins was used to perform a broad-scale proteomic analysis (Somalogic, Boulder, CO, USA) using their aptamer-based technology to quantitatively evaluate proteins present in the bone marrow aspirate between control mice and mice engrafted with AML samples. The SomaScan® assay quantitatively transforms the proteins present in a biological sample into a specific SOMAmer-based DNA signal. A SOMAmer-protein binding step is followed by a series of partitioning and wash steps that converts relative protein concentrations into measurable nucleic acid signals that are quantified using DNA detection technology, which for the SOMAscan assay with over 1,300 SOMAmer reagents is by hybridization to custom DNA microarrays. Assay details are provided in Gold et al. (2010).

#### RNA-sequencing analysis methods

FastQ files were generated using CASAVA BCL to FastQ (version 2.16). Sequencing yield was typically ∼25 million strand-specific paired-end reads. The RSEM package (version 1.2.29; Li and Dewey, 2011) in conjunction with the STAR alignment algorithm (version 2.5.1b; Dobin et al., 2013) was used for the mapping and subsequent gene-level counting of the sequenced reads with respect to mm10 Ensembl genes downloaded from the UCSC Table Browser 15 on 14th April 2016 (Karolchik et al., 2004). The “–forward-prob” parameter was set to “0” and all other parameters were kept as default. Normalization was performed with DESeq2 package (median of ratios method) (version 1.22.2; Love et al., 2014) with subsequent regularization using log_10_ transformation. Differential expression analysis was then performed with the DESeq2 package under the R programming environment (version 3.2.3) (R Development Core Team, 2015). Genes with FDR < 0.1 were judged to be differentially expressed. Genes from each given pairwise comparison were ranked using the Wald statistic. To analyze the transcriptome of stromal components in human xenografts, dimensionality of gene expression from AML, CB and ctrl samples was reduced with the t-distributed stochastic neighbor embedding (tSNE) technique for 2D visualization using the plot_tsne function from the bioplotr package on R. A comparison analysis was then performed between AML and ctrl samples in order to obtain differentially expressed genes. This analysis was performed using the contrast function within DESeq2 package. Linear correlation coefficients were calculated for control (ctrl) samples and a sample distance heatmap was then obtained using the heatmap package under R environment. Dimensionality reduction analysis was performed using gene expression data from ctrl samples. Principal component analysis (PCA) was made by using the expression matrix of normalized and regularized genes using the prcomp function on R. The results were visualized on a 3D map using the plot3d function from the rgl package on R. Multiple comparison of stromal populations in control samples was performed within the DESeq2 computing environment. We performed pairwise comparisons of all stromal cell types generating fold changes of each gene in each comparison of stromal types. Significant genes included in cluster analysis were selected by requiring at least one significant fold change in the pairwise stromal comparison (adjusted p value lower than 0.05). Clustering of selected genes was performed by applying a density-based algorithm (Rodriguez and Laio, 2014) where peaks in the distribution of z-scored expression across stromal types are associated to cluster centers. The pipeline adopted for this analysis consists of (1) fast search of density peaks in the distribution of normalized counts, (2) assignment of genes to clusters (density peaks) and (3) exclusion of noisy genes located in regions of low density. Cluster compactness was quantified as the average Pearson’s correlation between the mean cluster profile and the z-scored profiles of genes within the cluster. To obtain insights on how different cell types mediate interactions among pathways we applied a force-based visualization technique. First, we built a bipartite network with two node types for stromal cells and relevant molecular processes and edges weighted by the interactions described above. Second, we run a standard under-damped dynamic with elastic attraction and electric repulsion among nodes to find their equilibrium configuration. In order to characterize ctrl cluster 2 into sub-clusters of genes enriched in specific BM endothelial functions a database from Langen et al. (2017) was downloaded and compared against genes present in cluster 2. This dataset divides endothelial cells into three main categories, sinusoidal endothelium cells (type L), specialized capillary subtype (type H), and specialized endothelial cell subtype (type E). A heatmap was obtained using the pheatmap package on R with the option ward.D2 as a clustering method on row scaled data. To characterize genes present in ctrl cluster 1 specific to MSC functions, we performed a meta-analysis in which gene expression of leptin-receptor-positive (Lepr+) mesenchymal stromal cells from Tikhonova et al. (2019) were joined to the gene expression of Nes^low^, Nes^high^, and Ng2 samples from our study. All datasets were joined in a matrix and normalized together using DESeq2. Normalization and regularization were done as described before obtaining gene expression data for the different cell types. Comparisons were done for those genes present in cluster 1 and a heatmap was performed using the heatmap package on R with the option ward.D2 as a clustering method on row scaled data. For the global transcriptome analysis, significantly deregulated genes sharing similar deregulation patterns between control and leukemic samples were classified into groups by applying a model-based Bayesian framework. Deregulation patterns were represented by vectors of length equal to the number of stromal types, with elements taking discrete values: 0 (non-significant regulation), 1 (upregulation) and −1 (downregulation). We adapted the generative model introduced in Diana et al. (2019) to our data to detect deregulation patterns.

#### Secretome analysis

Data was analyzed using the Bioconductor version 2.13 (http://www.bioconductor.org) running on R version 3.0.2 (available from www.R.project.org). Protein intensity values were quantile normalized and log2 transformed. Differences in protein expression between groups was assessed using an empirical Bayes moderated t-statistics test from the “limma” package. P values were adjusted for multiple testing correction using the Benjamnini-Hocberg method. PCA was made by using the expression matrix of normalized and regularized genes using the prcomp function on R. The results were visualized on a 3D map using the plot3d function from the rgl package on R. Ligand-receptor pairs database was downloaded from Ramilowski et al. (2015) and compared against protein expression of the secretome from somascan arrays and gene expression from the statistically significant comparisons of AML versus ctrl RNaseq. Two lists of pairs for each cell type were obtained with the secretome as ligand and cell gene expression as receptor. To generate the dynamic figure, we used ligand-receptor pairs which were found coherently up or down- regulated in any of the stromal types. We constructed a bipartite graph representing cell-specific ligand-receptor connectivity and used force-driven dynamics on the network nodes to visualize the structure. We combined these results within a github (https://github.com/). The graphic map of the ligand-receptor interactome was sketch draw by adapting the thickness of the connection lines to the number of interactions between each cell type and the secretome, and between cell types.

### Quantification and statistical analysis

Information on number of replicates used in each experiment can be found in the figure legends. Details on OMICs analysis method and statistics can be found in Method details section. Based on sample size and intra-group variability in transcriptomic and proteomic data, significance was defined by an adjusted p value < 0.1

## Supplemental information

Document S1. Figures S1–S6

Table S1. Differentially expressed genes among BM niche populations, related to Figure 2 *Z* scores of genes associated to each cluster.

Table S2. Putative genes associated with MSC signature, related to Figure 2 Sub-clusters of cluster 1 enriched in MSC signature genes.

Table S3. Differentially expressed genes in BM niche populations upon AML engraftment, related to Figure 3 Top deregulated genes in the BM niche upon human AML engraftment.

Table S4. Differentially expressed proteins in the BM secretome upon AML engraftment, related to Figure 5 Top deregulated proteins in the BM secretome upon human AML engraftment. Proteins validated by comparison with clinical data are depicted

Table S5. Protein array raw normalized data, related to STAR Methods

Document S2. Article plus supplemental information
