# Supplementary material for: Integrated OMICs unveil the bone-marrow microenvironment in human leukemia
Source: Cell Rep. 2021 May 11;35(6):109119. doi: 10.1016/j.celrep.2021.109119 (PMC8131581; doi:10.1016/j.celrep.2021.109119)
Supplement: Document S1. Figures S1–S6 [file mmc1.pdf]

Cell Reports, Volume *35*

Supplemental information

Integrated OMICs unveil the bone-marrow microenvironment in human leukemia

Diana Passaro, Manuel Garcia-Albornoz, Giovanni Diana, Probir Chakravarty, Linda Ariza-McNaughton, Antoniana Batsivari, Clara Borràs-Eroles, Ander Abarrategi, Alexander Waclawiczek, Luigi Ombrato, Ilaria Malanchi, John Gribben, and Dominique Bonnet

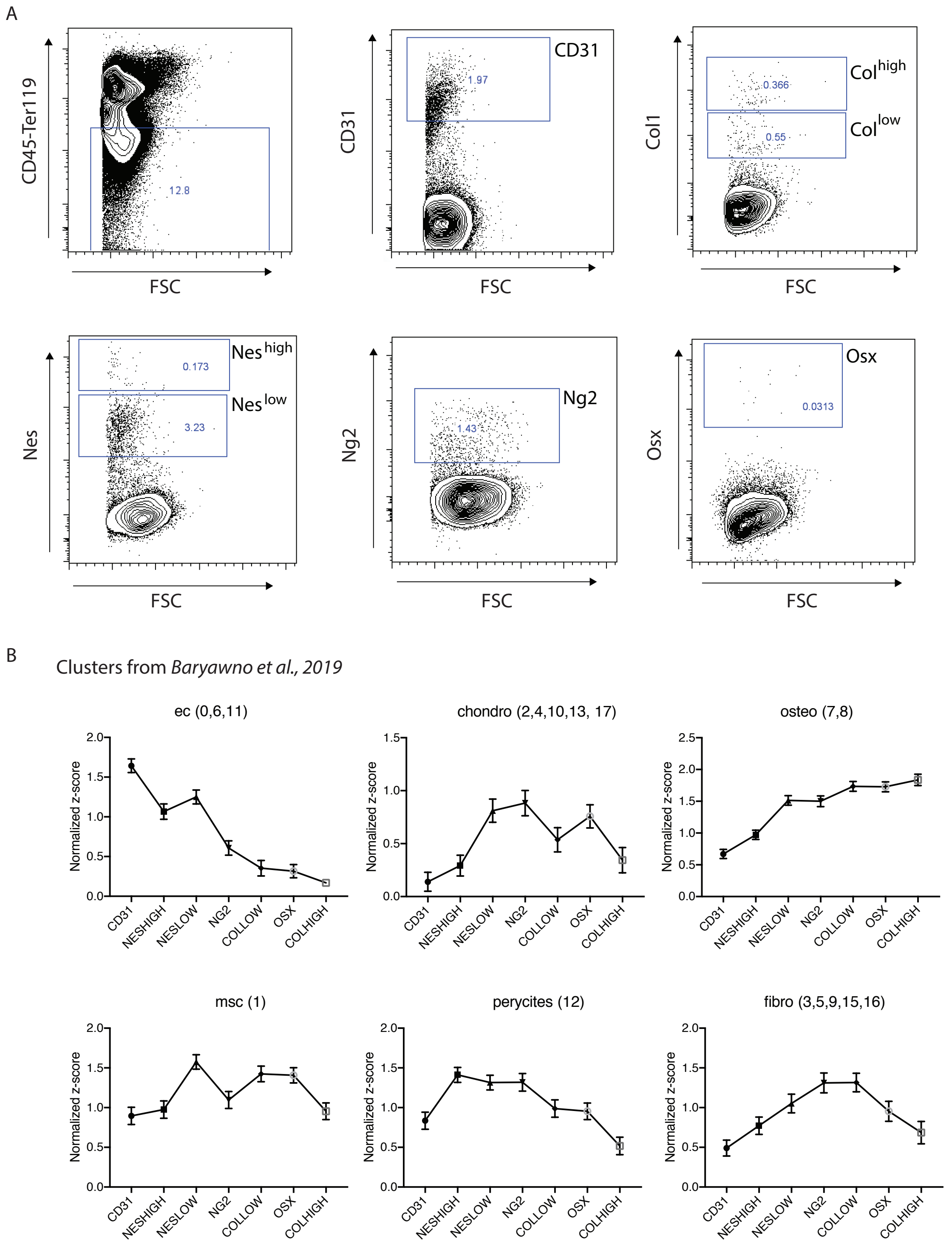

Figure S1. BM niche populations in homeostasis. (A) FACS gating strategy of BM niche cellular components. (B) Baryawno et al. clusters behaviors amongst niche components. Normalized z-scored across stromal types from control samples (means ± sem) is shown. Related to Figure 1 and 2.

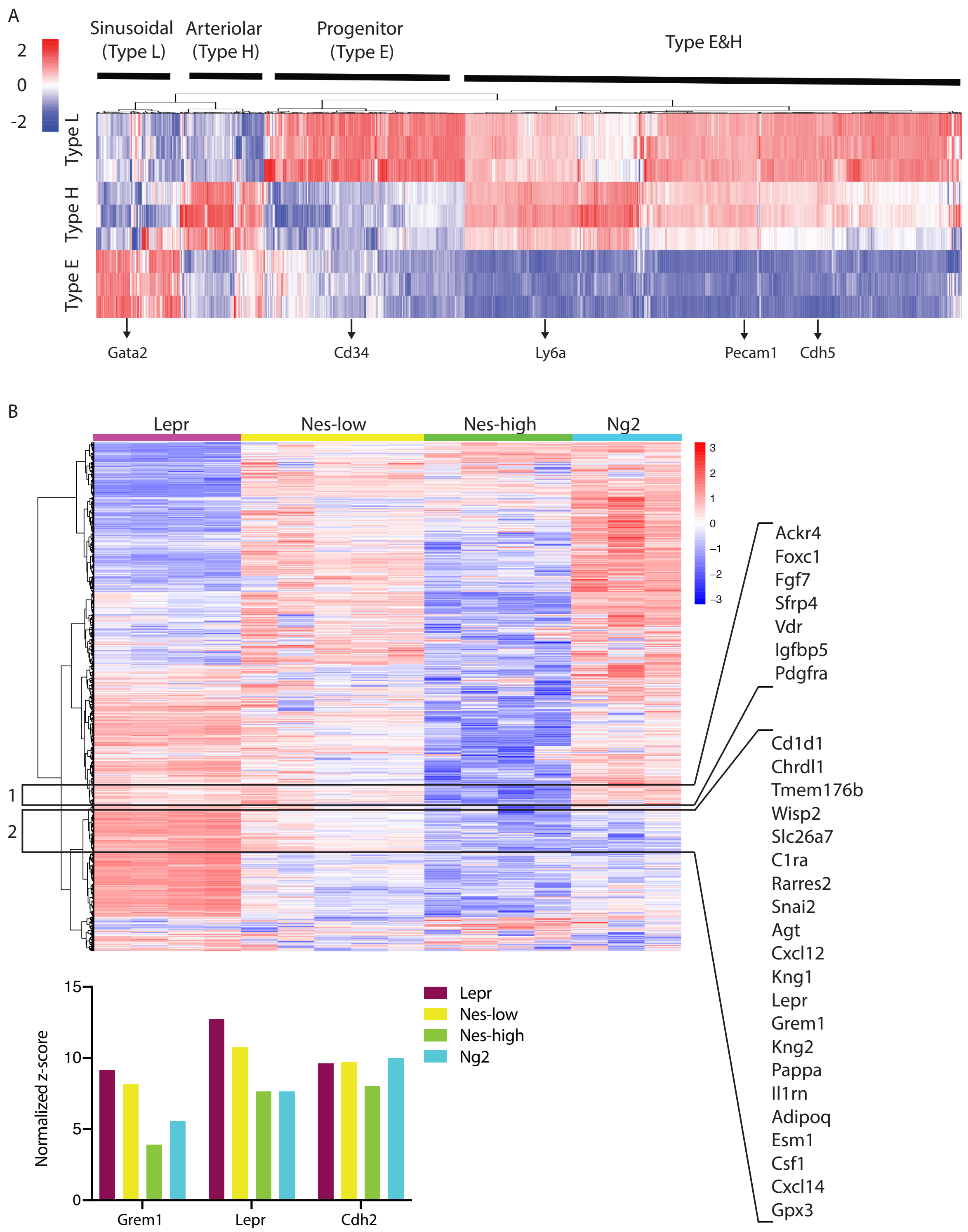

Figure S2. Differential clusters between BM niche populations. (A) Heatmap showing expression levels of genes in cluster 2 in different BM endothelial subpopulations. (B) Heatmap showing expression levels of genes in cluster 1 in different mesenchymal stromal population. Sub-clusters enriched for MSC signature genes are highlighted (see table S2 for details). Related to Figure 2.

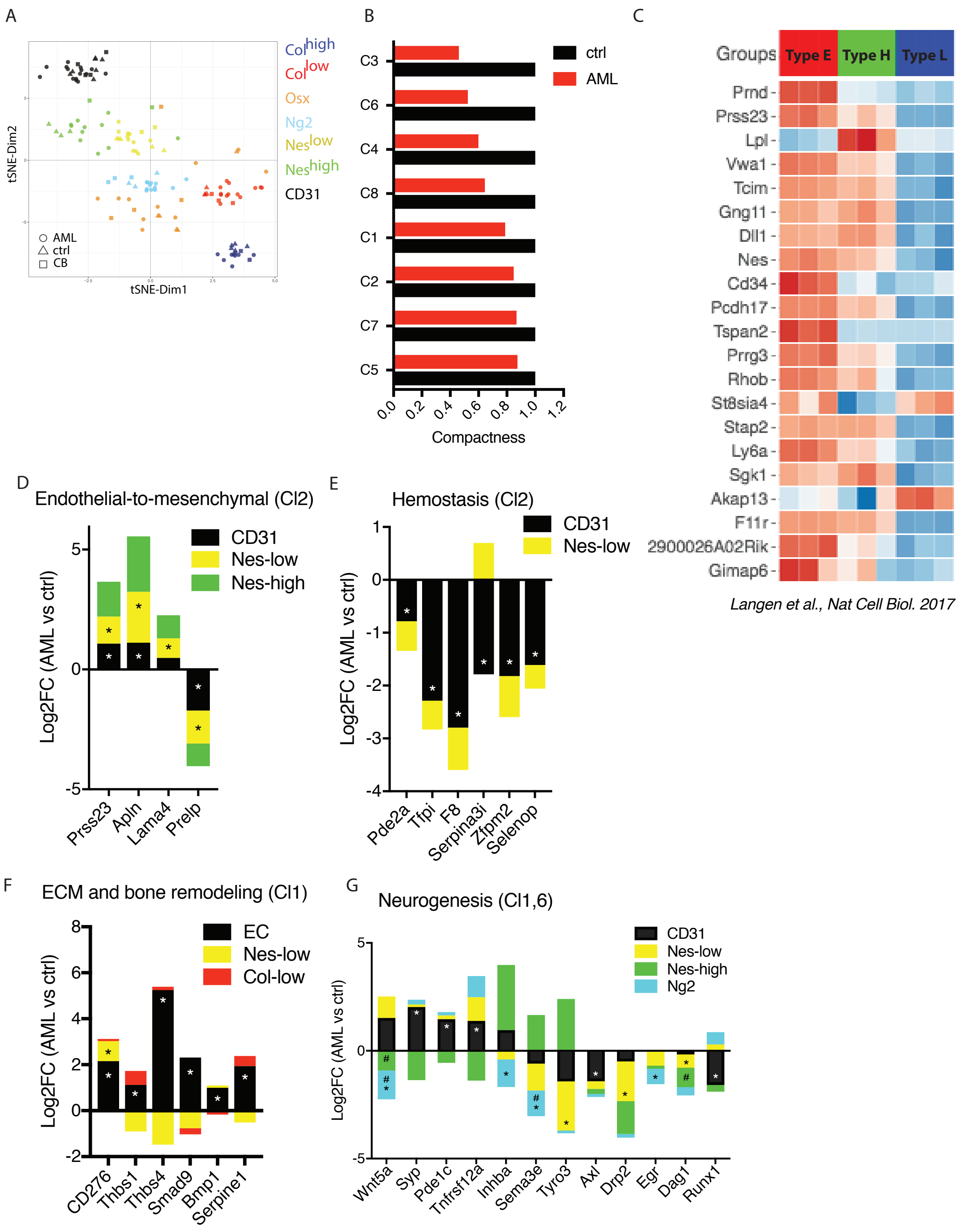

Figure S3. Human AML impact on BM niche homeostatic balance. (A) tSNE map of different stromal components in ctrl, healthy and AML xenografts. (B) Cluster compactness in ctrl (black) vs AML (red). (C) Heatmap showing expression levels of endothelial genes of cluster 2 unregulated in the presence of AML in different BM endothelial subpopulations. (D-G) Deregulation (mean log2FC) of genes in depicted clusters in the presence of AML (* means FDR < 0.1). Related to Figure 3.

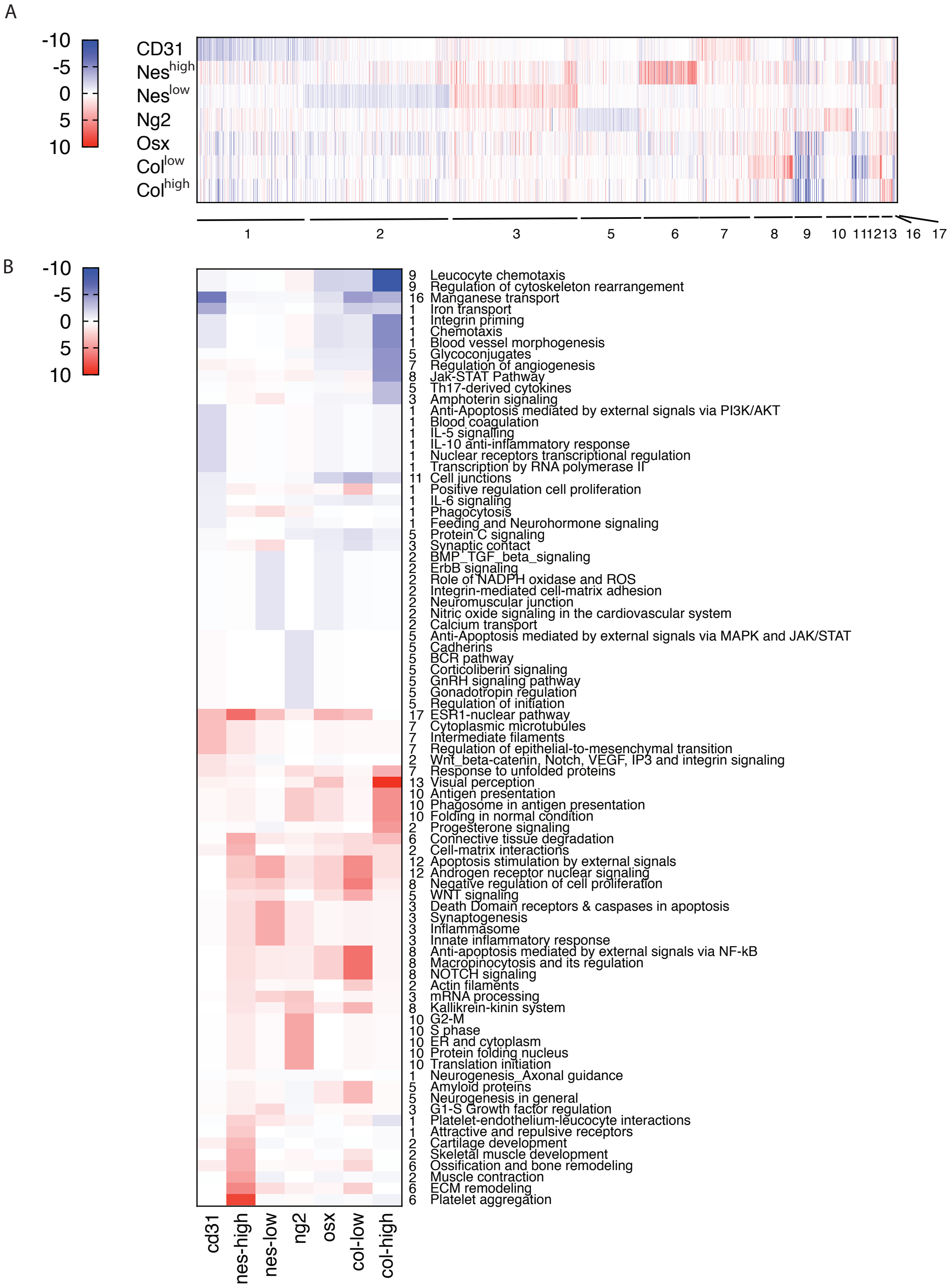

Figure S4. **Human AML impact on BM niche.** (A) Heatmap showing the log2FC of the top deregulated genes in each stromal cell types, clustered based on machine learning pattern aggregation algorithm (see methods for details). (B) Process map based on mean FC of each pattern. Related to Figure 3.

A

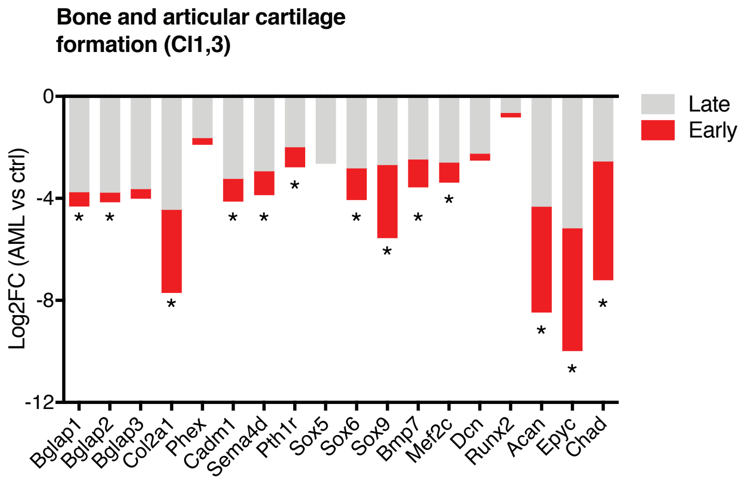

B

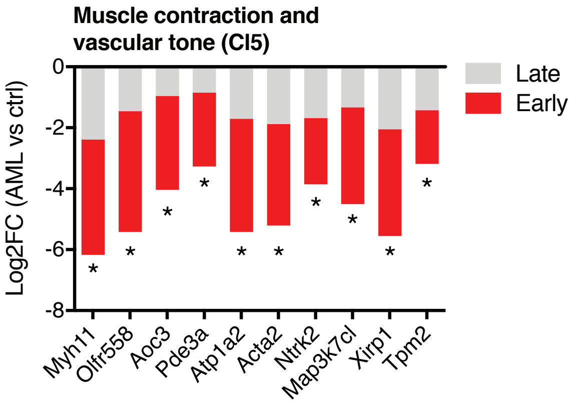

Figure S5. Progressive detriment of the BM mesenchymal niche in AML at early time points. (A, B) Deregulation (Mean log2FC) of genes in depicted clusters in the presence of AML in Late and Early stage of leukemic engraftment in mesenchymal niche (Nes^{low} cells) (* means FDR < 0.1 and are depicted only for Early). Related to Figure 4.

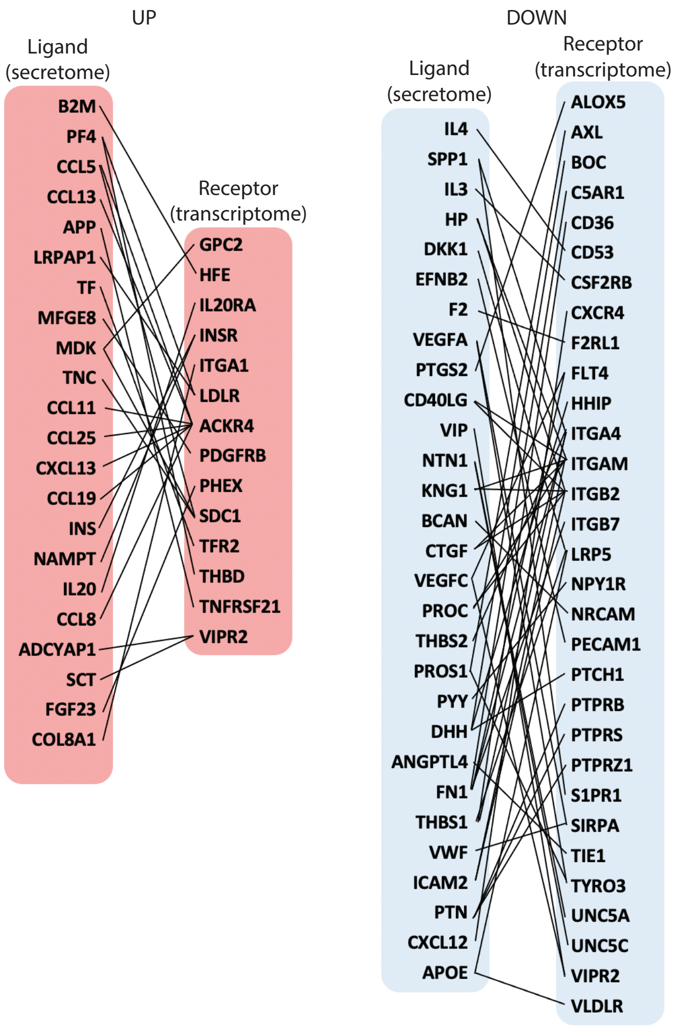

Figure S6. Integration of niche transcriptome and BM secretome. Sketch graphs of upregulated (red) and downregulated (blue) predicted ligand-receptor interactions between BM secretome and niche transcriptome. Related to Figure 6.
